# Lithium protects against paraquat neurotoxicity by NRF2 activation and miR-34a inhibition in SH-SY5Y cells

**DOI:** 10.3389/fncel.2015.00209

**Published:** 2015-05-28

**Authors:** Begum Alural, Aysegul Ozerdem, Jens Allmer, Kursad Genc, Sermin Genc

**Affiliations:** ^1^Izmir Biomedicine and Genome Center, Dokuz Eylul UniversityIzmir, Turkey; ^2^Department of Neuroscience, Health Science Institute, Dokuz Eylul UniversityIzmir, Turkey; ^3^Department of Psychiatry, School of Medicine, Dokuz Eylul UniversityIzmir, Turkey; ^4^Department of Molecular Biology and Genetics, Izmir Institute of TechnologyUrla, Turkey

**Keywords:** lithium, bipolar disorder, Parkinson's disease, NRF2, miR-34a

## Abstract

Lithium is a mood stabilizing agent commonly used for the treatment of bipolar disorder. Here, we investigated the potential neuroprotective effect of lithium against paraquat toxicity and its underlying mechanisms *in vitro*. SH-SY5Y human neuroblastoma cells were treated with paraquat (PQ) 0.5 mM concentration after lithium pretreatment to test lithium's capability in preventing cell toxicity. Cell death was evaluated by LDH, WST-8, and tryphan blue assays. Apoptosis was analyzed using DNA fragmentation, Annexin V immunostaining, Sub G1 cell cycle analysis, and caspase-3 activity assays. BCL2, BAX, and NRF2 protein expression were evaluated by Western-blotting and the BDNF protein level was determined with ELISA. mRNA levels of *BCL2, BAX, BDNF*, and *NRF2* target genes (*HO-1, GCS, NQO1)*, as well as miR-34a expression were analyzed by qPCR assay. Functional experiments were done via transfection with NRF2 siRNA and miR-34a mimic. Lithium treatment prevented paraquat induced cell death and apoptosis. Lithium treated cells showed increased anti-apoptotic protein BCL2 and decreased pro-apoptotic protein BAX expression. Lithium exerted a neurotrophic effect by increasing BDNF protein expression. It also diminished reactive oxygen species production and activated the redox sensitive transcription factor NRF2 and increased its target genes expression. Knockdown of NRF2 abolished neuroprotective, anti-apoptotic, and anti-oxidant effects of lithium. Furthermore, lithium significantly decreased both basal and PQ-induced expression of miR-34a. Transfection of miR-34a specific mimic reversed neuroprotective, anti-apoptotic, and anti-oxidant effects of lithium against PQ-toxicity. Our results revealed two novel mechanisms of lithium neuroprotection, namely NRF2 activation and miR-34a suppression.

## Introduction

Lithium is a mood-stabilizing agent widely used for treatment of acute episodes as well as for long-term relapse prevention in patients with bipolar disorder (BD), an illness with evident abnormalities in brain pointing at a neurodegenerational process or represent disturbed neuronal development (Cousins and Grunze, 2012; Diniz et al., 2013; Malhi et al., 2013). Apart from mood stabilization, neuroprotective and neurotrophic effects of lithium have been identified in clinical, preclinical, and *in vitro* studies (Forlenza et al., 2014). A wide variety of *in vivo* and *in vitro* studies confirmed neuroprotective effect of lithium against various insults (Pasquali et al., 2010; Diniz et al., 2013), thus rendering a potential for lithium in treatment of chronic neurodegenerative disorders such as Alzheimer's disease, Parkinson's disease (PD), Amyotrophic lateral sclerosis, and Huntington's disease.

Lithium's mechanism of action is still not clear; however, it is known to modulate neurotransmitter release, reduces oxidative stress and apoptosis, and induces angiogenesis, neurogenesis, and neurotrophic responses (Forlenza et al., 2014). Lithium exerts its biological effects via multiple signaling pathways. Inhibition of glycogen synthase kinase 3beta (GSK-3β) and inositol monophosphatase (IMPase), decreased the expression of the pro-apoptotic protein BAX and increased the expression of the anti-apoptotic protein BCL-2, activation of the cell survival kinases and increased expression of such neurotrophic factors brain-derived neurotrophic factor (BDNF) are well-known effects of lithium (Forlenza et al., 2014).

Paraquat (PQ) is widely used as an herbicide to control weeds (Moretto and Colosio, 2013; Goldman, 2014). Several epidemiologic studies suggest that the subacute exposure to PQ increases the incidence rate of PD in humans (Jenner et al., 2013; Goldman, 2014). In addition, PQ administration to rodents induces various features of PD, including motor deficits, dopaminergic neuronal loss and α-synuclein aggregation (Blesa et al., 2012). Therefore, PQ toxicity may be considered as a useful model to study dopaminergic cell death associated with PD. The definite mechanism of PQ neurotoxicity is not fully understood. However, several mechanisms have been implicated such as mitochondrial complex I inhibition and increase in reactive oxygen species (ROS) formation (Moretto and Colosio, 2013).

Oxidative stress plays a major role in the degeneration of dopaminergic neurons in PD (Dias et al., 2013). One of the neuroprotective strategies may be to prevent oxidative stress via activation of antioxidant defense systems. The nuclear factor erythroid 2-related factor 2 (NRF2) is a key transcription factor that activates anti-oxidant response element (ARE) containing anti-oxidant genes including heme oxygenase-1 (*HO-1*), peroxiredoxin (*PRX*), NAD(P)H:quinone oxidoreductase 1 (*NQO1*), glutamatecysteine ligase (*GCL*), and glutathione peroxidase (*GPX*) (Milani et al., 2013; Gan and Johnson, 2014). NRF2 deficiency increases MPTP sensitivity in mice whereas NRF2 overexpression protects against MPTP toxicity (Chen et al., 2009b).

MicroRNAs (miRNAs), are short, single stranded, endogenous, non-coding RNAs which have been discovered in 1993 (Lee et al., 1993). They regulate gene expression by targeting the 3′ UTR region of their target mRNAs via degradation or translational repression (Stroynowska-Czerwinska et al., 2014). MicroRNAs can control most biological processes including development, cell cycle, apoptosis, differentiation, and angiogenesis (Tüfekci et al., 2014). Several miRNAs exhibiting brain-specific expression and have been reported to be associated with physiological and pathological processes in the brain (Sun and Shi, 2014). MiRNA microarray studies showed that several miRNAs expression altered in PD patients' brain and blood samples (Miñones-Moyano et al., 2011; Vallelunga et al., 2014; Serafin et al., 2015). Peripheral miRNAs expression changes could be used for differential diagnosis of PD from Multiple System Atrophy (Vallelunga et al., 2014). Additionally, Anti-parkinsonian drugs alter blood miRNAs expression (Serafin et al., 2015). Besides, neurotoxins such as PQ cause miRNA expression changes in neuronal cells (Narasimhan et al., 2014). Additionally, lithium regulates the expression of some miRNAs and these may play a role in lithium's neuroprotective effects (Chen et al., 2009a).

In the present study we examined the protective effect of lithium on PQ neurotoxicity and its mechanisms in human SH-SY5Y cell line.

## Materials and methods

### Cell culture

SH-SY5Y cells were maintained in Dulbecco's Modified Eagle Medium: Nutrient Mixture F-12 (DMEM:F12) (Gibco, Gaithersburg, MD) supplemented with heat-inactivated fetal bovine serum (10% v/v), L-glutamine (1% v/v) and penicillin-streptomycin (1% v/v) at 37°C in 5% CO_2_.

### Lactate dehydrogenase release assay

The effects of lithium on PQ-induced cytotoxicity were measured by LDH release assay using Cytotoxicity Detection Kit^PLUS^ (Roche Diagnostics, Germany) according to the manufacturer's protocol.

### WST-8 assay

Cell viability was assessed by the ability of viable cells to reduce WST-8 reagent. Following treatment, 10 μL of WST-8 reagent were added to each well-containing 100 μL of medium and the plate was incubated for 4 h at 37°C. Afterwards, the light absorbance of each well was measured on a microplate reader (Biotek, USA) at 450 nm. Cell viability was expressed as a percentage of the untreated cells.

### Trypan blue staining

Cell death was also evaluated by trypan blue staining. After incubation, cells were stained with trypan blue stain for 1 min, and stained cells were photographed by using an inverted microscope (Olympus IX41, Japan).

### DNA fragmentation assay

Cell Death Detection ELISA Kit was used to evaluate DNA fragmentation in the cytoplasm of apoptotic cells. The data are expressed as fold increase in optical density as compared with control treated cells.

### Measurement of sub-G1 DNA content

At the end of treatment, cell pellets were resuspended in DNA staining reagent containing 50 μl RNAse (50 μg/ml) and 450 μl propidium iodide (100 μg/ml) and incubated for 15 min at room temperature. A total of 10,000 cells in each sample were analyzed and the percentage of apoptotic cells accumulated in the sub-G1 peaks were determined by using the FACS Canto II analyzer (Becton Dickinson, USA).

### Annexin V immunostaining

Annexin V-FITC apoptosis detection kit I (Becton Dickinson, USA) was used to detect apoptotic cells. After staining procedure, cells were fixed with 4% paraformaldehyde for 20 min. Stained cells were photographed by using an inverted microscope (Olympus BX50, Japan).

### Caspase-3 activity

Caspase-3 activity was measured as an indicator of apoptosis using colorimetric caspase activity assay kit (Invitrogen, USA) according to manufacturer's protocol.

### Intracellular ROS quantification

After 24 h lithium (2 mM) incubation period, supernatant was discarded and the cells were incubated with 100 μl PBS containing 10 μM CM-H_2_DCFDA for 1 h. Afterwards, CM-H_2_DCFDA was removed from each well and cells were treated with PQ (500 μM) or both PQ (500 μM) and lithium for 24 h. Fluorescence (at 520 nm) of dichlorofluorescein (DCF) in the supernatant was measured in response to excitation at 492 nm by using a microplate reader (Varioskan, Thermo).

### Quantitative PCR for mRNAs

Total RNA was isolated by using the Nucleospin RNA II Kit (Macherey-Nage, Germany) and converted to cDNA with RevertAid First Strand cDNA Synthesis Kit (Thermo Fermentas, ABD). Real-time quantitative PCR (qPCR) was performed using the Lightcycler 1.5 Instrument (Roche Diagnostics, Germany) and the SYBR-Green I kit according to the manufacturer's instructions. The primers used in the qPCR reactions are listed in Supplementary Table 1. The relative expression levels of mRNAs were quantified by using the 2^−ΔΔ^ Ct method.

### Western blot analysis

After treatment, cells were harvested, and total or fractionated protein was isolated from SH-SY5Y cells. Equal amounts of protein were resolved by 10–12% SDS–PAGE, transferred to a PVDF membrane and blocked with 3% BSA. Antibody dilution in immunoblotting was performed as follows: anti-NRF2 (1:250), anti-BCL-2 (1:500), anti-BAX (1:500), Lamin A/C(1:1000), and actin (1:1000). Immunodetection was measured using enhanced chemiluminescence (SuperSignal West Pico, Thermo Fisher Scientific, USA) according to the manufacturer's instructions. The resulting bands were analyzed with densitometer (UVP Gel Imager System, CA) and measured intensities of corresponding actin bands were used as cytoplasmic or total protein loading control while lamin A/C was used as nuclear loading control.

### Transfection for NRF2 siRNA

Small interfering RNA experiments were performed by using human-specific NRF2 or non-targeting siRNA (On-TargetPlus SMART pool human NFE2L2; Dharmacon). Cells were transfected with 50 nM siRNA, using Dharmafect reagent (Thermo Scientific), according to the manufacturer's instructions.

### Real-time PCR analysis of miR-34a

Total RNA was isolated from cells using Qiagen miRNeasy Mini Kit. Then, 2 μg RNA was reverse-transcribed using miScript II RT Kit (Qiagen, Valencia, CA). Real-time qPCR was performed using miScript SYBR Green PCR Kit on a Lightcycler® 480 Real-Time PCR System (Roche Diagnostics, Germany). Primer for mature miR-34a, SNORD95 and U6 were purchased from Qiagen (Valencia, CA). The relative expression levels of miRNAs were normalized to that of internal controls U6 and SNORD95 snRNA by using the 2^−ΔΔ^ Ct method.

### miR-34a target gene and pathways analysis

BDNF 3′UTRs were collected from ENSEMBL (Flicek et al., 2013) from its 10 available alternative versions which lead to 4 different 3′UTRs. RNA22 version 2 (Miranda et al., 2006), PITA (Kertesz et al., 2007), and TargetScan version 6 (Ruby et al., 2007) were used to predict miR-34a target binding sites within the 3′UTRs. Additionally, TarBase (Ruby et al., 2007) and miRTarBase (Hsu et al., 2011) were screened for miR-34a targets.

Reactome (D'Eustachio, 2011) was used for the pathway analysis of the miR-34a targets (4142 without duplicates) available on TarBase (477) and miRTarBase (4700) with BDNF added (Supplementary Table 2).

### Transfection with miRNA mimics and inhibitor

The miR-34a mimic, inhibitor and a negative control mimic were purchased from Qiagen. Transfections of cells with mimics (50 nM) were performed using HiPerFect Transfection Reagent (Qiagen, Valencia, CA). Total RNA from samples were isolated after 48 h of transfection for quantification of miRNA and target genes' expression.

### BDNF ELISA

The BDNF content in the culture medium was measured using BDNF ELISA Kit (Boster Biomedical, China) according to the manufacturer's protocol.

### Neurite outgrowth and neurite length

After transfection, serum-starved cells were treated with lithium and PQ and they were photographed with inverted phase-contrast microscope (Olympus CX41, Japan) at 40X magnification. All experiments were performed in triplicate. At least 30 neurites were analyzed for each sample. Neurite outgrowth was quantified by calculating the number of processes per cell. Neurite length was measured using the Image J software (NIH, USA) and expressed as mean process length. A neurite was defined as a cell process longer than 10 μm.

### Statistical analysis

Statistical analyses were performed with SPSS 18.0. Values represent the mean ± standard error of the mean (SEM). Comparison of two groups was analyzed by using the Mann-Whitney U test. P values smaller than 0.05 were considered to be statistically significant.

## Results

### Lithium protects SH-SY5Y cells from PQ-induced cell death

**Figure 1:**
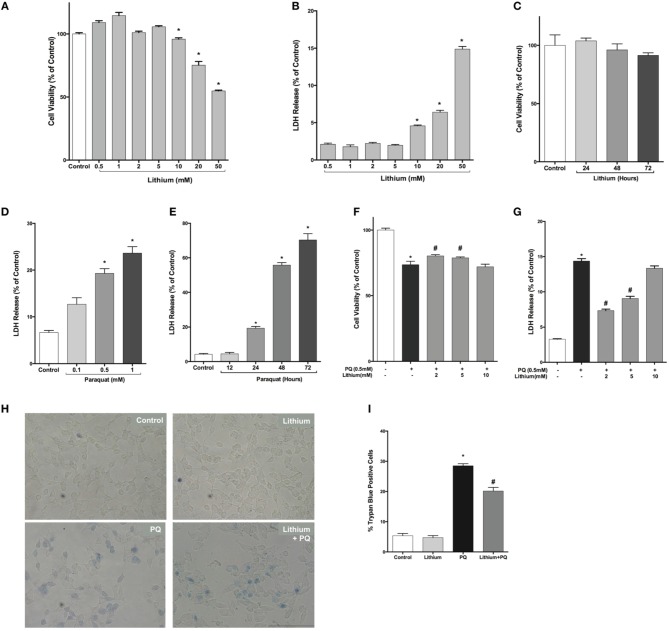
**Lithium decreases paraquat toxicity in SH-SY5Y cells**. Cells were treated with different doses of lithium (0.5–50 mM) for 24 h. Upon lithium treatment, **(A)** cell viability was quantified by WST-8 assay and **(B,C)** percentage of cell death was analyzed by LDH release assay. 10 mM and higher doses of lithium significantly reduced cell viability. PQ toxicity in SH-SY5Y cells is **(D)** dose and **(E)** time dependent. SH-SY5Y cells were treated with lithium (2–5 mM) for 24 h and then incubated with 0.5 mM PQ for a further 24 h. **(F)** Cell viability was quantified by WST-8 assay and **(G)** cell death was determined by LDH release assay. PQ treatment increased cell death and lithium reversed the toxic effect of PQ in SH-SY5Y cells. Representative light microscopy images **(H,I)** of trypan blue staining showed the neuroprotective effect of lithium against PQ-induced cell death. The data are presented as mean ± standard error (S.E), *n* = 5. (^*^p < 0.05 compared to control and ^#^*p* < 0.05 compare to PQ treated cells).

First, we examined lithium cytotoxicity by LDH and WST-8 assays. Concentrations of lithium up to 10 mM did not result in an increased rate of cell death, but higher doses (> 10 mM) of lithium were toxic to SH-SY5Y cells (Figures 1A,B). Lithium treatment at 2 mM concentration did not decrease cell viability during 24–72 h incubation periods (Figure 1C). PQ alone increased cell death in SH-SY5Y cells in a dose and time dependent fashion (Figures 1D,E).

Next, we evaluated lithium effect on cell viability. PQ treatment significantly decreased cell viability (73.6 ± 2.6%). Lithium pre-treatment (2–5 mM) increased cell viability to 80.3 and 78.9%, respectively (Figure 1F).

Lithium effect on PQ-induced cell damage was further investigated using LDH release assay. PQ treatment increased LDH release from SH-SY5Y cells (14.4 ± 0.3%). Pretreatment with 2–5 mM lithium reduced the LDH release to 7.3 ± 0.2 and 9.5 ± 0.3%, respectively (Figure 1G). However, 24 h pretreatment with 10 mM lithium had no effect on PQ-induced cytotoxicity.

The effect of lithium on cell death was further confirmed by trypan blue staining. PQ treatment significantly increased the percentage of cells stained with trypan blue (Figures 1H,I). On the contrary, lithium pretreatment significantly reduced the percentage of cells stained with trypan blue.

### Lithium decreases PQ-induced apoptosis in SH-SY5Y cells

**Figure 2:**
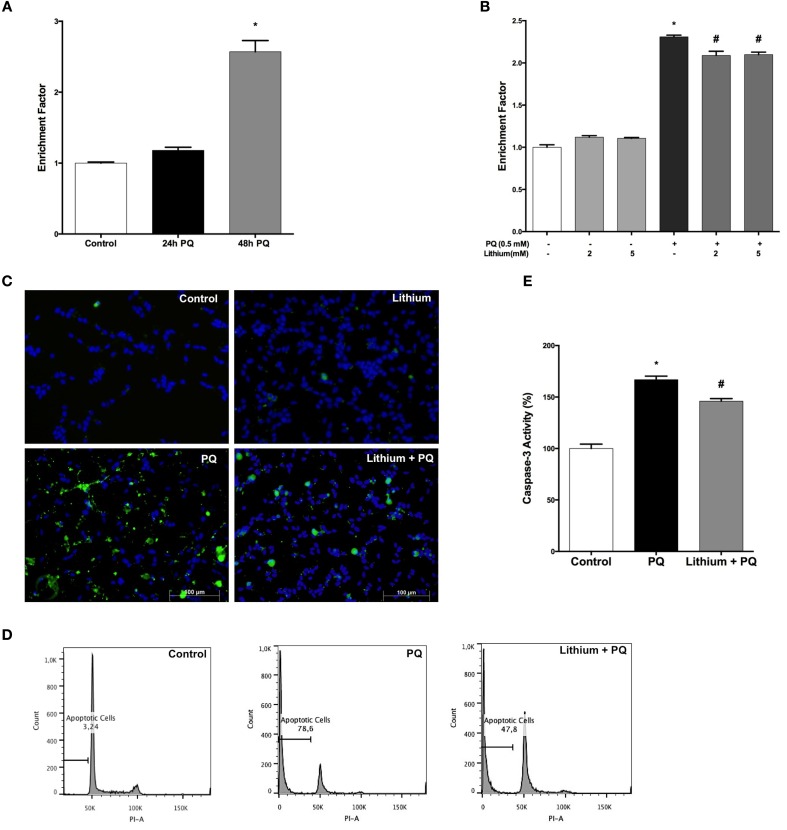
**Lithium reduces apoptotic cell death induced by PQ in SH-SY5Y cells. (A)** DNA fragmentation was increased with 0.5 mM PQ treatment, which was analyzed by Cell Death ELISA assay. **(B)** Lithium (2 mM and 5 mM) pretreatment reduces DNA fragmentation induced by PQ in SH-SY5Y cells. **(C)** Apoptotic cells were stained by Annexin-V-FITC dye and visualized using immunofluorescence microscopy. **(D)** Flow cytometric analysis of the sub G1 apoptotic population was assessed by using PI staining. Lithium attenuates PQ induced increase of sub G1 apoptotic population in SH-SY5Y cells **(E)**. Caspase-3 activity was evaluated in lysates of treated cells by spectrophotometric detection of the chromophore p-nitroaniline (pNA) formed after cleavage from the labeled substrate DEVD-pNA. Lithium reduced PQ induced caspase-3 activity increase in SH-SY5Y cells. The data are presented as mean ± S.E, *n* = 5. (^*^p < 0.05 compared to control and ^#^*p* < 0.05 compare to PQ treated cells).

Our results showed a significant 2.4-fold increase in DNA fragmentation upon 48 h of PQ treatment (Figure 2A). Pretreatment with lithium attenuated PQ induced DNA fragmentation significantly (Figure 2B).

Lithium effect on apoptosis was evaluated by Annexin-V immunostaining. PQ treatment markedly increased the annexin V-positive cells and this increase was prevented by lithium pretreatment (Figure 2C).

Next, we analyzed the effect of lithium on PQ-induced apoptosis by assessing the sub-G1 cells in PI-stained samples of SH-SY5Y cells by flow cytometry. We observed a significant increase in sub-G1 cells population (78.6%) after 48 h PQ treatment while treatment with 2 mM lithium decreased the ratio of sub-G1cells to 47.8% (Figure 2D).

We also measured the activity of caspase-3 as an indicator of apoptosis. PQ-treatment for 24 h at a dose of 0.5 mM significantly increased the caspase-3 activity. Lithium pretreatment prevented PQ-induced increase in caspase-3 activity (Figure 2E).

### Lithium reverses the expressions of BCL-2 family genes altered by PQ

**Figure 3:**
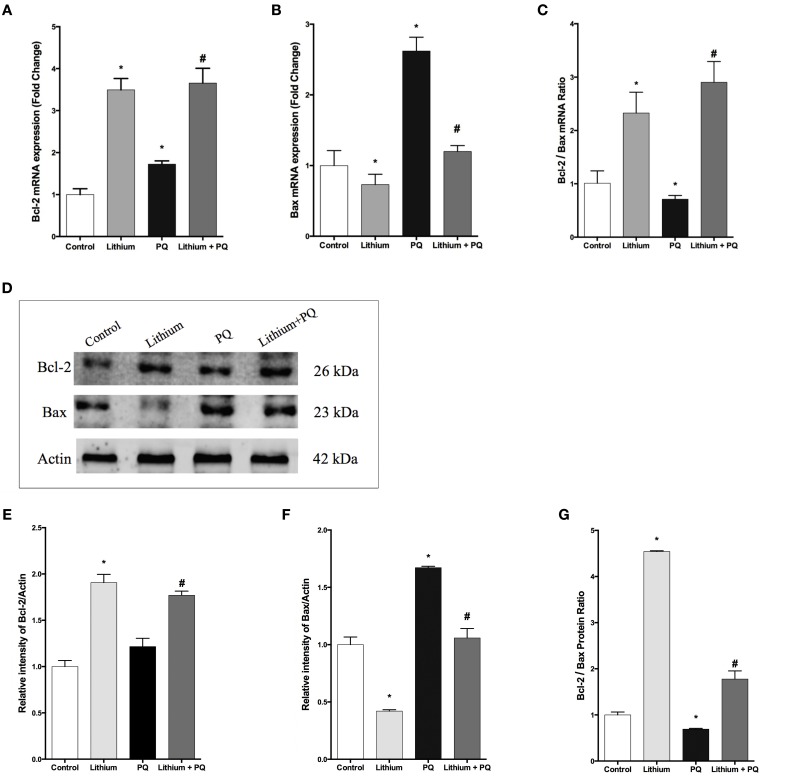
**Lithium modulates BCL-2 family genes levels in SH-SY5Y cells**. SH-SY5Y cells were pretreated with lithium (2 mM) for 12 h before 0.5 mM of PQ incubation. Intracellular **(A)** *BCL-2* and **(B)** *BAX* mRNA levels were quantified by qPCR. Both PQ and lithium treatment increased the *BCL-2* mRNA level. PQ treatment increased the *BAX* mRNA level and lithium pretreatment reversed PQ's effect on the *BAX* mRNA level. **(C)** Ratio of *BCL-2* to *BAX* mRNA levels was markedly reduced by PQ treatment and lithium pretreatment increased this ratio. Intracellular **(D,E)** BCL-2 and **(D,F)** BAX protein levels were analyzed by western blot and actin was used as a loading control. The intensity of proteins were quantified by using Image J (NIH Bethesda). **(G)** Ratio of BCL-2 to BAX was markedly reduced by PQ treatment whereas lithium pretreatment increased this ratio. The data are presented as mean ± S.E, *n* = 5. (^*^p < 0.05 compared to control and ^#^*p* < 0.05 compare to PQ treated cells).

As shown in Figure 3A, lithium treatment alone significantly increased *BCL-2* mRNA expression at 12 h. PQ treatment also increased *BCL-2* mRNA expression 1.7 fold compared to control cells. Additionally, lithium pretreatment resulted in further increase of *BCL-2* mRNA expression in SH-SY5Y cells.

The expression of *BAX* mRNA increased 2.6-fold after PQ treatment in SH-SY5Y cells. Lithium pretreatment reduced *BAX* mRNA expression induced by PQ. Lithium alone also decreased *BAX* mRNA expression in SH-SY5Y cells (Figure 3B).

In addition, the *BCL-2* / *BAX* mRNA expression ratio was markedly reduced upon PQ treatment while lithium pretreatment significantly increased this ratio (Figure 3C).

Consistent with gene expression analysis, lithium treatment increased BCL-2 protein level 1.9 fold, but PQ treatment did not alter the BCL-2 protein level in SH-SY5Y cells (Figures 3D,E). BCL-2 protein expression also increased in Lithium and PQ treated cells (Figures 3D,E). Lithium alone decreased BAX protein levels (0.42 fold) in SH-SY5Y cells. BAX protein level increased 1.7 fold after PQ exposure while lithium pretreatment prevented PQ induced BAX protein increase in SH-SY5Y cells (Figures 3D,F). Additionally, the BCL-2/ BAX protein expression ratio was markedly reduced upon PQ treatment while lithium pretreatment significantly increased this ratio (Figure 3G).

### Lithium alters PQ-associated decrease in BDNF mRNA expression and secretion

**Figure 4:**
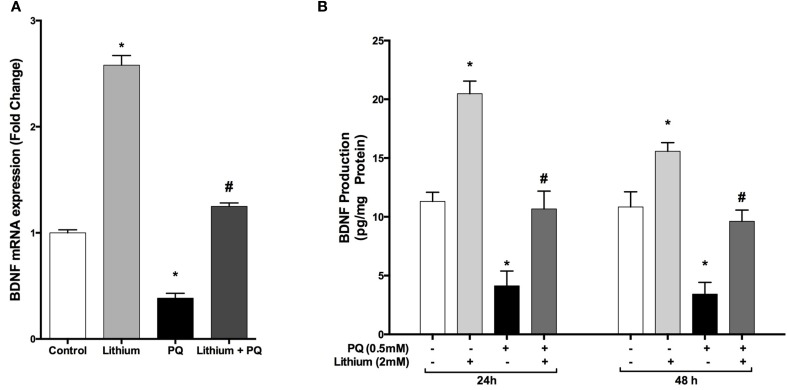
**Lithium increases BDNF expression and secretion in SH-SY5Y cells**. Cells were pretreated with lithium (2 mM) for 24 h prior to PQ exposure. **(A)** Intracellular *BDNF* mRNA levels were quantified by real time qPCR. PQ exposure markedly reduced the *BDNF* mRNA level while lithium pretreatment reversed the PQ effect on the *BDNF* mRNA level. **(B)** Secreted BDNF protein levels in culture medium were measured by BDNF ELISA kit. Lithium increased PQ induced BDNF decrease in SH-SY5Y cells. The data are presented as mean ± S.E, *n* = 5. (^*^p < 0.05 compared to control and ^#^*p* < 0.05 compare to PQ treated cells).

Our results showed that lithium alone increased *BDNF* mRNA expression by 2.7 fold whereas PQ treatment caused a 0.62-fold reduction in *BDNF* mRNA expression compared to control cells. Lithium pretreatment prevented the decrease in *BDNF* mRNA levels after treatment with PQ (Figure 4A).

Consistent with mRNA results, lithium treatment significantly increased BDNF secretion compared to control groups. Furthermore, PQ-associated decrease in BDNF levels was prevented by lithium pretreatment (Figure 4B).

### Lithium diminishes PQ-induced ROS production in SH-SY5Y cells

**Figure 5:**
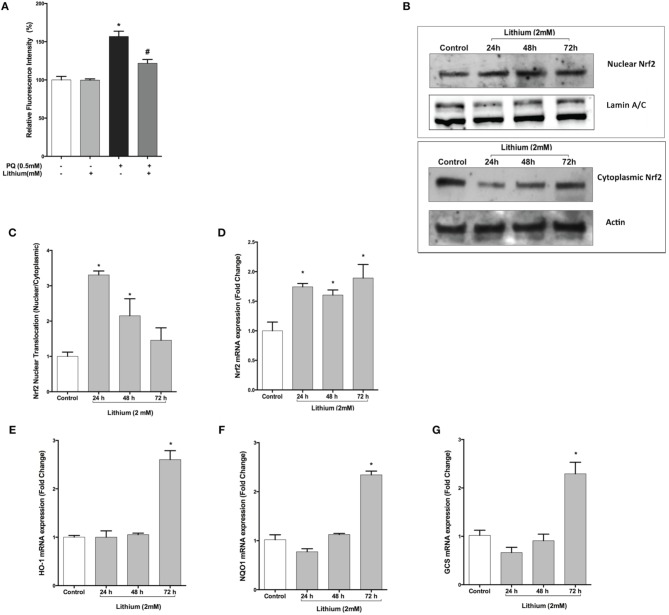
**Lithium reduces ROS production and activates the NRF2 pathway**. **(A)** SH-SY5Y cells were pretreated with lithium (2 mM) for 24 h then 0.5 mM of PQ was added. Intracellular ROS production was quantified by using the CM-H_2_DCFDA. Lithium reduced the PQ induced ROS production in SH-SY5Y cells. **(B)** Cells were treated with 2 mM lithium for 24, 48 and 72 h. Then, both nuclear and cytoplasmic NRF2 protein levels were analyzed by Western blot. Actin for cytoplasmic, lamin A/C for nuclear fractions were used as loading control. **(C)** The ratio of the integrated density of nuclear NRF2 to lamin A/C and cytoplasmic NRF2 to actin was quantified by using Image J (NIH Bethesda). Lithium increased nuclear translocation of NRF2. **(D)** *NRF2*, **(E)** *HO-1*, **(F)** *NQO1*, and **(G)** *GCS* gene expression levels were quantified by qPCR. Lithium treatment increased mRNA levels of NRF2 target genes. NRF2; nuclear factor erythroid 2-related factor 2, HO-1; heme-oxygenase-1, NQO1; NAD(P)H: quinone oxidoreductase, and GCS; glucosylceramide synthase. The data are presented as mean ± S.E, *n* = 5. (^*^p < 0.05 compared to control and ^#^*p* < 0.05 compare to PQ treated cells).

As shown in Figure 5A, PQ induced ROS production (156.8 ± 6.9 %) and pretreatment with lithium caused a significantly decrease ROS production induced by PQ (121.9 ± 4.9 %) compared to only PQ treated cells.

### Lithium triggers nuclear translocation and activation of NRF2 transcription factor in SH-SY5Y cells

Nuclear translocation of NRF2 transcription factor is considered to be an important mechanism for its activation. Western blot analysis demonstrated that translocation of NRF2 was significantly increased by 24 and 48 h lithium treatment (Figures 5B,C).

We then examined the effect of lithium on mRNA expression of *NRF2* and NRF2 -regulated genes including Heme-oxygenase-1 (*HO-1*), NAD(P)H: quinone oxidoreductase (*NQO1*), glucosylceramide synthase (*GCS*). As a shown in Figure 5D, The expression of *NRF2* mRNA was increased after 24 h lithium treatment and this increase was maintained up to 72 h. Furthermore, we found that 72 h lithium treatment increased mRNA levels of NRF2 target genes (Figures 5E–G).

### Knockdown NRF2 expression with siRNA inhibits its target genes' expression

**Figure 6:**
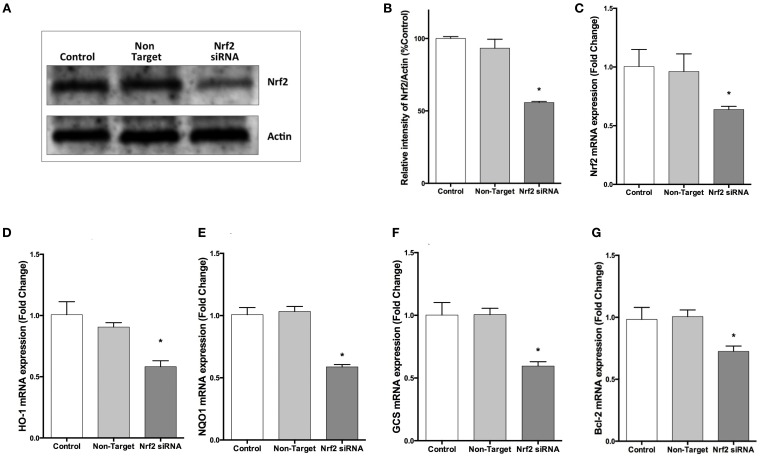
**NRF2 siRNA transfection decreases NRF2 and target genes mRNA and NRF2 protein expression in SH-SY5Y cells**. Cells were transfected with NRF2 siRNA or non-target siRNA for 24 h. **(A,B)** NRF2 protein levels were measured by Western blot, **(C)** mRNA levels of *NRF2* were evaluated with qPCR. Transfection of cells with NRF2 siRNA decreased *NRF2* mRNA and protein expression. Knocking-down NRF2 expression reduced the mRNA expression of **(D)** *HO-1* and **(E)** *NQO1* **(F)** *GCS* and **(G)** *BCL-2* determined by qPCR. The data are presented as mean ± S.E, *n* = 5. (^*^p < 0.05 compared to non-target siRNA transfected cells).

To confirm the repression by NRF2 specific siRNA, we evaluated *NRF2* mRNA expression by qPCR and NRF2 protein expression by Western blotting. Transfection of cells with NRF2 siRNA decreased the NRF2 expression at both mRNA and protein levels (Figures 6A–C). In addition, NRF2 inhibition significantly reduced the mRNA expression of NRF2-regulated genes, *HO-1, NQO1, GCS*, and *BCL-2* compared to cells transfected with non-targeting siRNA (Figures 6D–G).

### NRF2 knockdown reverses neuroprotective, anti-apoptotic and anti-oxidant effects of lithium on PQ-induced cell death

**Figure 7:**
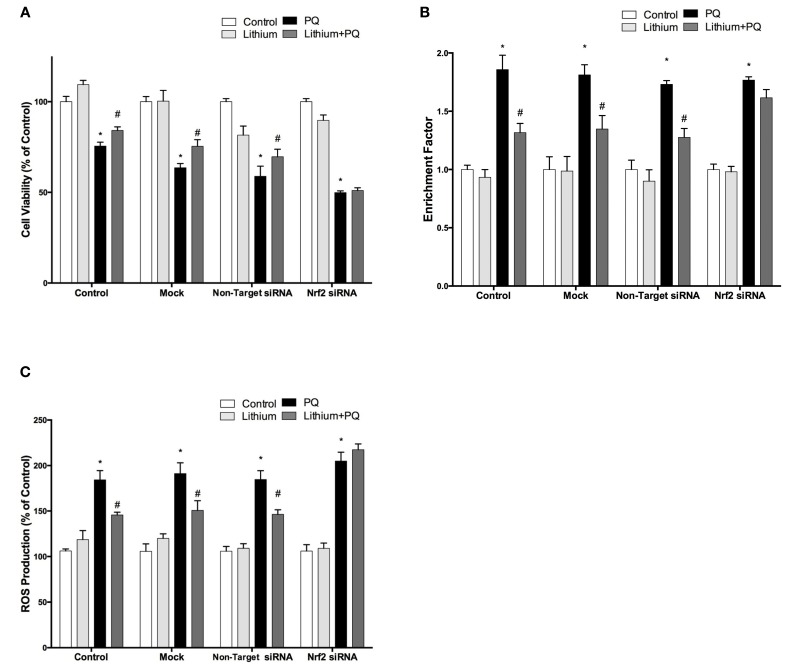
**Down-regulation of NRF2 reversed the neuroprotective and anti-oxidant effects of Lithium**. Cells were transfected with NRF2 siRNA or non-target siRNA. Upon transfection, cells were pretreated with 2 mM lithium prior to 0.5 mM PQ treatment. **(A)** Cell viability, **(B)** Apoptosis and **(C)** ROS production were quantified via WST-8 assay, Cell Death ELISA and CM-H_2_DCFDA method, respectively. Neuroprotective and anti oxidant effects of lithium were reversed by NRF2 siRNA transfection. The data are presented as mean ± S.E, *n* = 5. (^*^p < 0.05 compared to control and ^#^*p* < 0.05 compare to PQ treated cells).

We further investigated whether the neuroprotective, anti-apoptotic, and anti-oxidant effects of lithium were abolished by siRNA knockdown of NRF2. After transfection, the effect of lithium on cell viability, apoptosis, and ROS production were examined with WST-8 assay, DNA fragmentation and CM-H_2_DCFDA, respectively. We found that neuroprotective, anti-apoptotic, and anti-oxidant effects of lithium were abolished by silencing NRF2 expression with specific siRNA transfection (Figures 7A–C). These findings suggest that NRF2 activation has a critical role for the neuroprotective, anti-apoptotic, and anti-oxidant effects of lithium.

### The effect of lithium and NRF2 knockdown on neurite outgrowth

**Figure 8:**
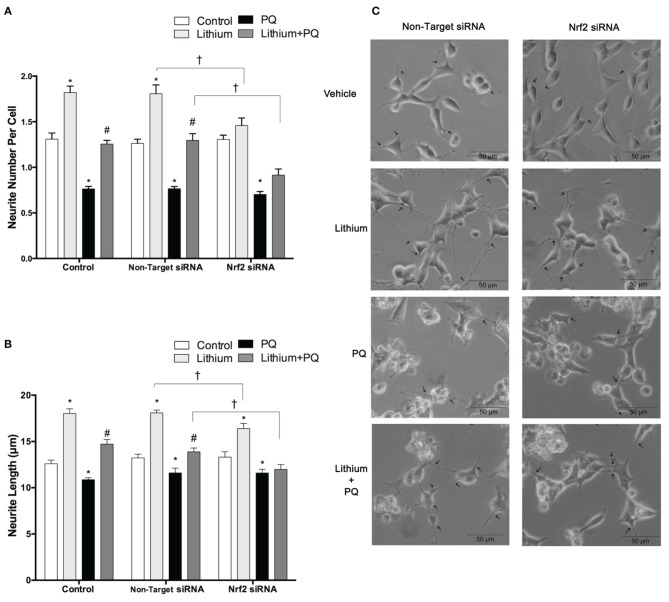
**Lithium may exerts neurite outgrowth effect via Nrf2 activation**. After NRF2 siRNA or non-target siRNA transfection, SH-SY5Y cells were treated with 2 mM lithium for 24 h prior to 0.5 mM PQ treatment. **(A)** Neurite cell number was counted using ImageJ 1.42 (http://imagej.nih.gov/ij/). **(B**) The average maximal neurite length for both, the transfected and non-transfected cells were analyzed by ImageJ 1.42 (http://imagej.nih.gov/ij/). **(C**) Representative phase-contrast microscopy images are shown in order to indicate neurite outgrowth distribution. Arrowheads indicate the neurites arising from cells. Briefly, lithium pretreatment increased neurite numbers and length which decreased by PQ in non-transfected and non-target siRNA transfected groups. Nrf2 knockdown partially reversed the effects of lithium on neurite outgrowth. At least 30 neurites were analyzed for each sample. Scale bar, 50 μm. The data are presented as mean ± S.E, *n* = 3. (^*^p < 0.05 compared to control, ^#^*p* < 0.05 compared to PQ treated cells and ^†^*p* < 0.05 compared to Non-Target siRNA).

Lithium significantly increased neurite outgrowth in SH-SY5Y cells. To investigate the role of NRF2 on lithium-mediated neurite outgrowth, SH-SY5Y cells were transfected with NRF2 siRNA. We found lithium treatment increased neurite number per cell and neurite length in control cells and non-target siRNA transfected cells. PQ treatment alone decreased neurite outgrowth, whereas lithium pretreatment reversed this decrease to the control levels (Figures 8A–C). However, the effect of lithium on neurite outgrowth was diminished in NRF2 siRNA transfected cells in both basal and PQ treated conditions. These results suggested that NRF2 could be involved in the lithium effect on neurite outgrowth in SH-SY5Ycells.

### Lithium downregulates miR-34a expression induced by PQ

**Figure 9:**
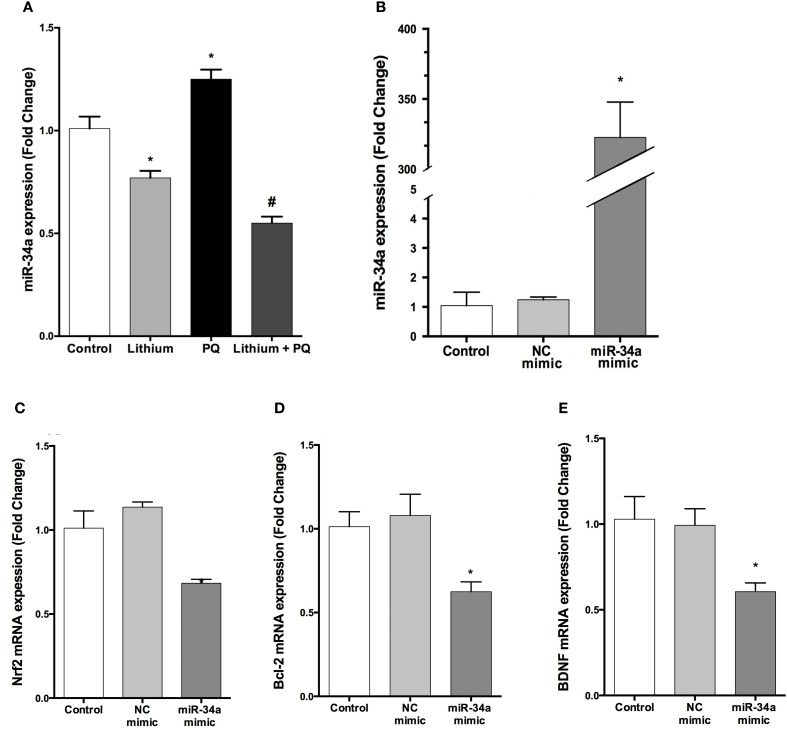
**Lithium decreases miR-34a expression and miR-34a overexpression significantly downregulates the expression of miR-34a target genes in SH-SY5Y cells. (A)** Cells were incubated with lithium or PQ and expression levels of miR-34a were quantified by qPCR. Lithium treatment decreased miR-34a expression. SH-SY5Y cells were transfected with miR-34a mimic or negative control (NC) mimic for 48 h and the cells were collected for RNA isolation. **(B)** qPCR was performed for miR-34a to determine the efficiency of transfection and **(C–E)** mRNA levels of miR-34a target genes after transfection. miR-34a overexpression down-regulates *BCL-2* and *NRF2* and *BDNF* mRNA levels in SH-SY5Y cells. Control: cells without any transfection. The data are presented as mean ± S.E, *n* = 5. (^*^p < 0.05 and ^#^*p* < 0.05 compared to PQ treated cells).

We analyzed the expression of miR-34a by using qPCR. Our results indicate that lithium treatment alone decreased miR-34a expression compared to control cells. In addition, PQ treatment significantly increased miR-34a expression and lithium pretreatment prevented PQ induced increase of miR-34a expression (Figure 9A).

### The effects of up and down regulation of miR-34a expression on its target genes' expression

The transfection efficiency of mature miRNAs was assessed by qPCR. Our results showed that transfection with miR-34a mimic robustly upregulated miR-34a level 322.5-fold (Figure 9B).

To determine whether overexpressed miR-34a can downregulate its target genes expression, we investigated the mRNA levels of *BCL-2, NRF2*, and *BDNF* (a predicted target of miR-34a). Over-expression of miR-34a significantly reduced the mRNA levels of *BCL-2, NRF2*, and *BDNF* in SH-SY5Y cells (Figures 9C–E).

miR-34a antagomir treatment significantly inhibited basal mir-34a expression (Supplementary Figure 1A). On the other hand, miR-34 antagomir treatment did not cause any significant increase in BDNF mRNA and protein expression (Supplementary Figures 1B,C).

### The result of miR-34a target gene and pathways analysis

Mir-34a is an intergenic miRNA located on human chromosome 1 between the coding regions for GPR157 and HBPD. Neither TarBase, nor miRTarBase list BDNF among the many experimentally confirmed targets of miR-34a. Also, RNA22 and TargetScan did not return any binding sites for miR-34a within the 3′UTR of BDNF, suggesting that it does not affect BDNF expression levels. However, PITA predicted a borderline interaction (Score: −9.44) between miR-34a-5p and BDNF (ENST00000499568, ENST00000500662, and ENST00000502161) and another weaker (Score: −7.17), but still potentially relevant interaction with BDNF ENSEMBL transcripts ENST00000439476, ENST00000525528, and ENST00000395986 (Supplementary Table 3). But, the results of loss-of-function experiment did not point to a direct microrna-target interaction between miR-34a and BDNF.

**Figure 10:**
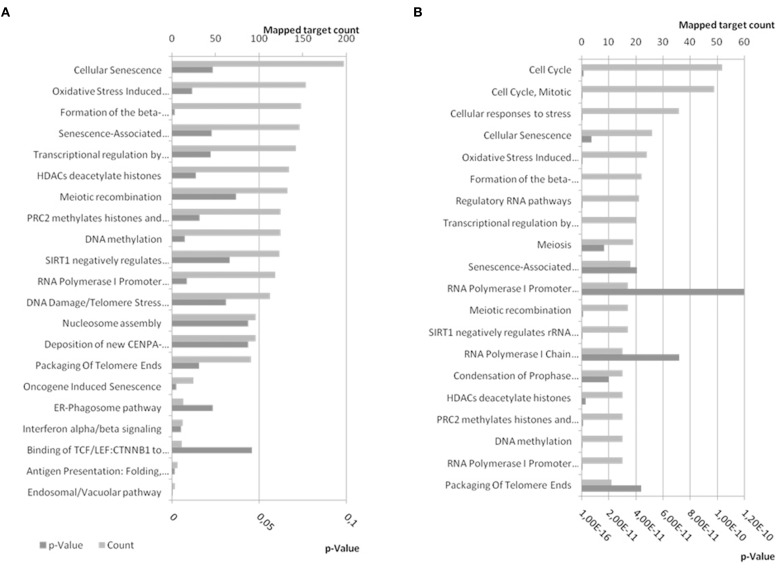
**Reactome pathways enriched in targets of miR-34a-5p**. **(A)** Experimentally confirmed miR-34a-5p targets from miRTarBase and TarBase were combined and BDNF was added. The target list was submitted to pathway analysis using EBI's Reactome. **(B)** Confirmed targets of miR-34a-5p shared between miRTarBase and TarBase and therefore likely more confident, were submitted to Reactome for pathway analysis. Bottom x-axis show the expected probability for pathway enrichment and upper x-axis shows the number of targets mapped to the given pathway. Dark gray bars refer to *p*-values whereas patterned gray bars indicate the number of mapped targets. In **(A)** pathways were selected to be below a *p*-value of 0.05 (21) whereas the first 20 pathways ordered by *p*-value were selected for **(B)**. A comprehensive list can be found in Supplementary Table 2. The enriched pathways are similar for combined and shared targets from miRTarBase and TarBase.

The 4142 non-duplicate identifiers, and 6 synonyms for BDNF, of miR-34a-5p targets were submitted to Reactome analysis and 1440 targets were used while Reactome was not able to map 2248 of them including BDNF and all its synonyms (Supplementary Table 2). Some of the pathways enriched in targets of miR-34a-5p have *p*-values below the general significance threshold of 0.05 and were selected for display (Figure 10A). The number of mapped targets for Cellular Senescence is largest (197) followed by Oxidative Stress (153). If only the targets shared between TarBase and miRTarBase (237) and BDNF and its synonyms are submitted to Reactome, a similar result is achieved with Cellular Senescence and Oxidative Stress having high prominence (Figure 10B).

### Over-expression of miR-34a reverses the neuroprotective and anti-apoptotic effects of lithium

**Figure 11:**
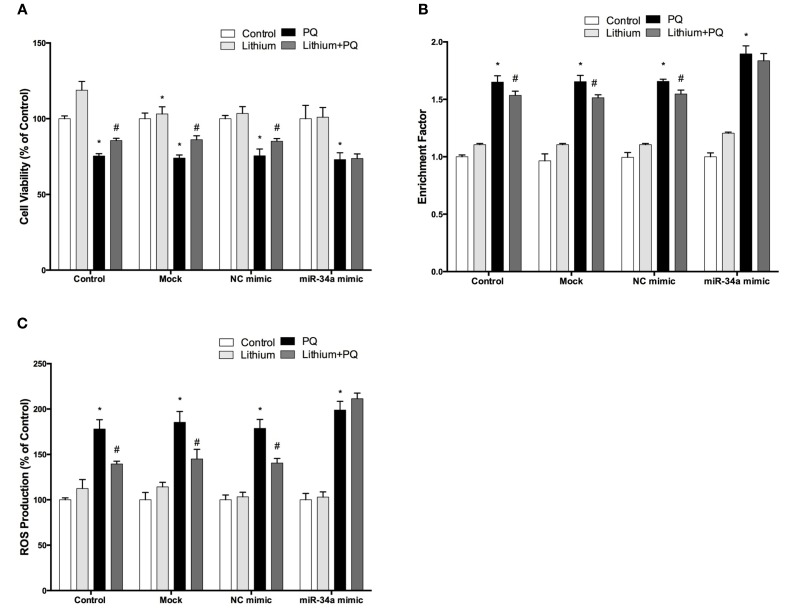
**Lithium may exert its neuroprotective, anti-apoptotic and anti-oxidant functions via miR-34a**. Mimic transfected (miR-34a mimic or negative control mimic) and non-transfected cells were treated with 2 mM lithium for 24 h prior to 0.5 mM PQ treatment. **(A)** Transfection of miR-34a mimic diminished the neuroprotective effect of lithium against PQ-toxicity. Overexpression of miR-34a also reversed the **(B)** anti-apoptotic and **(C)** anti-oxidant effects of lithium on SH-SY5Y cells. (^*^p < 0.05 compared to control and ^#^*p* < 0.05 compared to PQ treated cells).

To investigate the effect of miR-34a on lithium mediated neuroprotection in SH-SY5Y cells, we used miR-34a mimic to simulate overexpression of miR-34a. As shown Figure 11A, lithium significantly increased cell viability and this increase remained unchanged in negative control mimics transfected cells. Neuroprotective effect of lithium was reversed by up-regulation of miR-34a levels with mimic transfection in SH-SY5Y cells.

The lithium-mediated anti-apoptotic effect was also significantly reduced in the miR-34a mimic transfected cells as compared with cells that were transfected with a control mimic (Figure 11B). Our results indicate the important role of miR-34a in mediating the neuroprotective effect of lithium.

### Up-regulation of miR-34a expression reverses the anti-oxidant effect of lithium

We further investigated the role of miR-34a on the anti-oxidant effect of lithium. SH-SY5Y cells were transfected with miR-34a or control mimics, then the effects of lithium on the production of ROS was evaluated using CM-H_2_DCFDA. The lithium-mediated antioxidant effect was significantly reduced in miR-34a mimic transfected cells as compared with cells that were transfected with control mimics (Figure 11C).

### Over-expression of miR-34a abolish neurite outgrowth effect of lithium in SH-SY5Y cells

**Figure 12:**
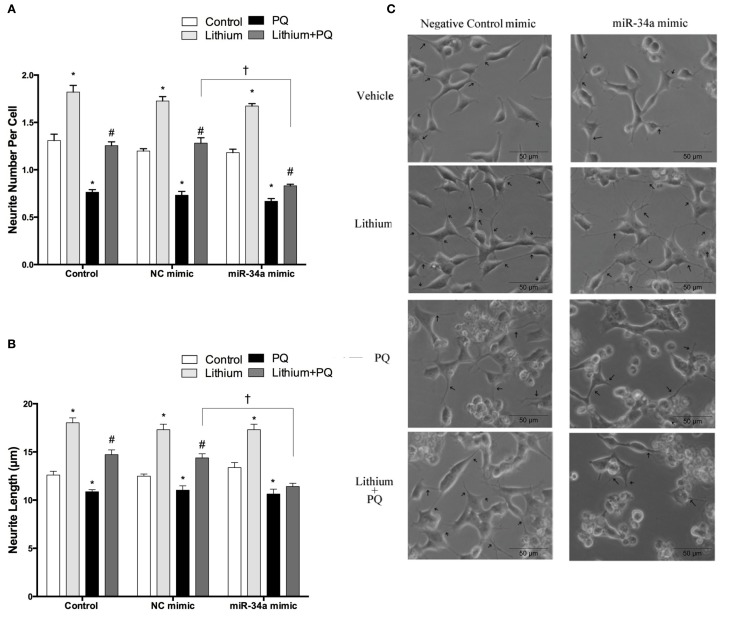
**Lithium may enhance neurite outgrowth via the regulation of miR-34a**. After transfection with mimic miR-34a, SH-SY5Y cells were treated with 2 mM lithium for 24 h prior to 0.5 mM PQ treatment. **(A)** Neurite cell number was counted using ImageJ 1.42 (http://imagej.nih.gov/ij/). **(B**) The average maximal neurite length for both, the transfected and non-transfected cells were analyzed by ImageJ 1.42 (http://imagej.nih.gov/ij/). **(C)** Representative phase-contrast microscopy images are shown in order to indicate neurite outgrowth distribution. Arrowheads indicate the neurites arising from cells. Lithium pretreatment reversed PQ induced decrease of neurite length. miR-34a mimic abolished lithium effect on neurite length. At least 30 neurites were analyzed for each sample. Scale bar, 50 μm. The data are presented as mean ± S.E, *n* = 3. (^*^p < 0.05 compared to control, ^#^*p* < 0.05 compared to PQ treated cells and ^†^*p* < 0.05 compared to NC mimic).

Neurite outgrowth was evaluated after transfection with mimics to determine the impact of miR-34a on lithium-mediated neurite outgrowth. While PQ treatment decreased neurite outgrowth, lithium alone significantly increased neurite number per cell and length. Lithium pretreatment prevented PQ induced decrease of neurite outgrowth. This effect of lithium remained unchanged in negative control mimic transfected cells. However, miR-34a mimic significantly reduced the neurite outgrowth effect of lithium (Figures 12A–C).

## Discussion

In the present study, the neuroprotective effect of lithium on PQ toxicity was evaluated in SH-SY5Y cells.

We first investigated lithium effect on cell viability to find optimum concentration of lithium without cytotoxicity. Previous studies determined that lithium concentrations of up to 4 mM for 4 days did not decrease viability of SH-SY5Y cells (Allagui et al., 2009). Additionally, lower dosage (approximately 2.3 mM) of lithium for longer periods (up to 5 days) has no cytotoxic effect in PC12 cells, a rat pheochromocytoma cell line (Yang et al., 2014). We chose to use 2 mM concentration of lithium at different time points (24–72 h) for further experiments.

Many recent studies have shown that exogenous environmental toxins, such as PQ are linked to PD (Tufekci et al., 2011; Baltazar et al., 2014). Here, we show that PQ is significantly toxic to SH-SY5Y cells in a time and dose dependent manner which is consistent with a recent study (Martins et al., 2013). PQ exposure leads to neuronal cell death *in vitro* and *in vivo;* though exact mechanisms of selective neurodegeneration induced by PQ in dopaminergic neurons are still unknown (Berry et al., 2010). In our study, lithium prevented PQ-induced neuronal cell death and increased cell viability at concentrations of 2–5 mM. To our knowledge, there is no study investigating protective effect of lithium in PQ induced neuronal death. The neurotrophic effect of lithium is well-documented in various cultured neuronal cell systems *in vitro*. In contrast to neurotoxins, lithium induces neurite outgrowth and differentiation of various neuronal cells. Lithium treatment of hippocampal neurons for 5 days increased dendritic outgrowth at 1–2 mM concentration (Park et al., 2014). In our study, lithium enhanced neurite number and length as markers of neurite outgrowth in SHSY5Y cells. We found PQ reduced secretion and mRNA levels of BDNF one of the main regulators of synaptogenesis and dendritogenesis (Nelson and Alkon, 2014). Lithium pretreatment rescued *BDNF* mRNA and secreted protein level. BDNF is a main partner for the neurotrophic effects of lithium. Postmortem studies showed that the BDNF level is decreased in the brain of PD patients especially in the substantia nigra region (Allen et al., 2013). PQ administration to mice also reduces BDNF level in the hippocampus region of the brain (Litteljohn et al., 2011). Treatment of rat cortical neurons with lithium for 6 days induced protein expression of BDNF (Hashimoto et al., 2002). Chronic treatment of rats with lithium for 14–28 days also increased BDNF expression in the hippocampus and temporal cortex (Fukumoto et al., 2001).

There is powerful evidence in human studies to support the role of oxidative stress in PD. It has been demonstrated that alteration of oxidative stress markers in serum and cerebrospinal fluid samples of PD patients (Buhmann et al., 2004). Post-mortem studies showed an increase in ROS production and a reduction in antioxidants such as glutathione in dopaminergic neurons (Dias et al., 2013). Deficiencies of mitochondrial complex I in the brain have also been shown in post-mortem PD studies (Subramaniam and Chesselet, 2013). Mutations in PD causing genes including parkin, α-synuclein, PTEN induced putative kinase 1, and DJ-1 also cause mitochondrial dysfunction leading to excessive production of ROS (Blandini, 2013; Guo et al., 2013; Zuo and Motherwell, 2013). PQ, as a redox cycling agent, exerts its cytotoxic effect by generating ROS including superoxide radical, hydrogen peroxide, and hydroxyl radicals and inhibits mitochondrial Complex I and Complex III function (Blanco-Ayala et al., 2014). PQ induced oxidative stress also leads to mitochondrial dysfunction. Here, as a measure of mitochondrial functioning, WST-8 assay showed PQ induced mitochondrial dysfunction which was significantly reversed by lithium pretreatment. Consistent with a previous study (Cristóvão et al., 2009), our findings also confirmed that PQ treatment induced oxidative stress in SH-SY5Y cells. Lithium pretreatment reversed PQ induced ROS production as shown by DCF analysis. Following the various pathogenic mechanisms such as mitochondrial dysfunction, oxidative stress, dysregulated calcium metabolism, misfolded protein responses, endoplasmic reticulum stress, and dysregulated autophagy, the final common step in neurodegenerative diseases is neuronal cell death. Apoptosis is the dominant mode of cell death in PD (Ghavami et al., 2014). Lithium's anti-apoptotic action is well-known and responsible for its therapeutic effect (Chiu et al., 2014). Lithium treatment increased ratio of anti-apoptotic to pro-apoptotic BCL-2 gene family members in lithium responders patients with bipolar disorder (Lowthert et al., 2012). In our study, PQ treatment induced apoptotic cell death and lithium pretreatment attenuated PQ induced apoptosis. We examined the mRNA and protein expression of BCL-2 and BAX, respectively by qPCR and Western blotting. It has been shown that PQ increases p53 protein and its pro-apoptotic target gene, BAX (Yang and Tiffany-Castiglioni, 2008). In contrast to this, p53 overexpression decreases BCL-2 expression (Song et al., 2012). We found that PQ decreased BCL-2/BAX ratio both in gene and protein expression level and lithium reversed these changes. Furthermore, lithium significantly decreased caspase-3 activity induced by PQ. Altogether, these results confirm that anti-apoptotic effect of lithium plays a significantly role in cytoprotection elicited by this drug against PQ neurotoxicity *in vitro*.

To elucidate the regulatory mechanisms of lithium's anti-oxidant effect at the transcriptional level, we evaluated its effect on gene expression of NRF2, a master modulator of redox homeostasis, and its target genes, namely *HO-1, NQO1*, and *GCS* (Gan and Johnson, 2014). NRF2 exerts anti-oxidant, cytoprotective and anti-inflammatory effects via targeting a set of battery genes, which bear ARE binding sites in their promoter regions (Gan and Johnson, 2014). In SH-SY5Y cells, PQ decreases protein levels of NRF2 and its target GCS (Yang et al., 2010). We found that lithium increased mRNA expression of NRF2 and its target genes. In addition, lithium promoted basal levels of NRF2 in both cytosolic and nuclear fractions in SH-SY5Y cells. Furthermore, lithium increases nuclear/cytosolic ratio of NRF2 suggests that it significantly induces translocation of NRF2 into the nucleus. To study the possible role of NRF2 in lithium's effects, we knocked down NRF2 gene using specific siRNA. The abolishing effect of *NRF2* gene silencing on lithium's neuroprotective and anti-oxidant effects in PQ toxicity suggest that they are likely dependent on the transcription factor NRF2.

Lithium may also exert its protective effect against PQ via post-trancriptional gene regulation. MicroRNAs have recently emerged as important mediators of post-trancriptional gene regulation (Bhalala et al., 2013). Here, we studied miR-34a, an apoptomir, mitomir, and redoximir which targets NRF2 and BCL-2. miR-34a level was found to be increased in postmortem cerebellar tissue from BD patients (Bavamian et al., 2015). Lithium altered seven miRNAs including miR-34a in 20 lymphoblastoid cell lines derived from BD patients (Chen et al., 2009a). Combined lithium and valproate treatment also decreased miR-34a level in cerebellar granule cells (Hunsberger et al., 2013). In addition, 4 week lithium treatment downregulated miR-34a expression in rat hippocampus (Zhou et al., 2009). miR-34a induces apoptosis by targeting anti-apoptotic genes such as BCL2. It also causes cell cycle arrest by silencing Cyclin-dependent kinase 4 (*CDK4*) and Cyclin-dependent kinase *(CDK6)* genes and decreases cell survival (Agostini and Knight, 2014). Moreover, NRF2 and its target genes thioredoxin and thioredoxin reductase 2 are targeted by miR-34a, thus leading to impairment of anti-oxidant defense system (Bai et al., 2011; Silva-Adaya et al., 2014). We found that PQ alone increased the basal expression of miR-34a. The possible reason of this increase may be the activation of miR-34a inducers nuclear factor of kappa light polypeptide gene enhancer in B-cells 2 (NFkB) and p53 transcription factors by PQ (Yang and Tiffany-Castiglioni, 2008; Yang et al., 2010; Kauppinen et al., 2013). Here, lithium significantly decreased both basal and PQ-induced expression of miR-34a. Currently, the mechanisms of lithium's effect on miR-34a expression are unknown, however, different mechanisms including activation of various transcription factors and signaling pathways or epigenetics factors such as DNA methylation may be responsible (Chiu et al., 2013; Agostini and Knight, 2014).

Next, we performed functional studies by transfection of miR-34 specific mimic and evaluated cell viability, neurotrophism, apoptosis, and ROS production. mRNA expression of two validated targets of miR-34a, *NRF2* and *BCL-2* significantly decreased with mimic transfection (Wang et al., 2009; Smith-Vikos and Slack, 2012). In contrast to the gain-of-function experiment, loss-of-function with antagomir did not show discordance between mir-34a expression and BDNF mRNA/protein levels. Although BDNF is predicted with low and minimum score in only one database, this prediction was not experimentally validated in our hands. Additionally, the results of functional experiment with miR-34a antagomir did not support the results of overexpression experiment. Since miRNA overexpression represents a supraphysiological condition, discordance between expression of a miRNA and its predicted target may not point to a functional miRNA-target interaction (Thomson et al., 2011). A plausible explanation for these results is that miR-34a overexpression may indirectly inhibit BDNF expression by suppressing the expression of CREB (one of its validated targets), which transactivates BDNF mRNA in normal conditions (Nair and Vaidya, 2006; Chang et al., 2011).

In addition to its classical use in BD, neurodegenerative diseases such as PD have been proposed as other indications for lithium therapy. We revealed two novel mechanisms for lithium neuroprotection, namely NRF2 activation and miR-34a suppression. The first mechanism represents an action at the transcriptional level and the second one point to a posttranscriptional effect. One may propose several other different mechanisms of neuroprotection provided by lithium. GSK-3β that activates Fyn kinase and this kinase results in nuclear export and degradation of NRF2 (Niture et al., 2014). Lithium inhibits GSK-3β activation and keeps NRF2 in the nucleus. NRF2 activation may inhibit miR-34a suppressing its inducer NFkB transcription factor (Stefanson and Bakovic, 2014).

In conclusion, since no proven medical treatment is currently available to slow disease progression, there is a great need for more effective novel therapeutic agents in the treatment of PD. Lithium may be a strong candidate drug for the treatment of PD. Here, we demonstrated the neuroprotective effect of lithium against PQ. Additionally, we revealed two novel mechanisms mediating lithium's effect on neurodegeneration.

### Conflict of interest statement

The authors declare that the research was conducted in the absence of any commercial or financial relationships that could be construed as a potential conflict of interest.
