# Supplementary material for: Lithium protects against paraquat neurotoxicity by NRF2 activation and miR-34a inhibition in SH-SY5Y cells
Source: Front Cell Neurosci. 2015 May 28;9:209. doi: 10.3389/fncel.2015.00209 (PMC4446540; doi:10.3389/fncel.2015.00209)
Supplement: Supplementary file 1 [file DataSheet1.DOCX]

| **GENE** | **Primer Sequences** |
| --- | --- |
| *GAPDH* | Forward: 5’-ACC ACA GTC CAT GCC ATC AC-3’  Reverse: 5’- TCC ACC ACC CTG TTG CTG TA -3’ |
| *BCL-2* | Forward: 5’- CTG GTG GAC AAC ATC GCT CTG -3’  Reverse: 5’- GGT CTG CTG ACC TCA CTT GTG -3’ |
| *BAX* | Forward: 5’- TGG TTG CCC TTT TCT ACT TTG-3’  Reverse: 5’-GAA GTA GGA AAG GAG GCC ATC -3’ |
| *BDNF* | Forward: 5’- AGC CTC CTC TGC TCT TTC TGC TGG A -3’  Reverse: 5’- CTT TTG TCT ATG CCC CTG CAG CCT T-3’ |
| *NRF2* | Forward: 5’- GCC TAG CAC AAG TAC CAC TCT TGG TC-3’  Reverse: 5’- CTG AGG CAG GAG AAT TGC TGG AAC C-3’ |
| *HO-1* | Forward: 5’- GAG ACG GCT TCA AGC TGG TGA TG-3’  Reverse: 5’- GTT GAG CAG GAC GCA GTC TTG G-3’ |
| *GCS* | Forward: 5’- GCC TAG CAC AAG TAC CAC TCT TGG TC -3’  Reverse: 5’- CTG AGG CAG GAG AAT TGC TGG AAC C -3’ |
| *NQO1* | Forward: 5’- TTG ATT AAG GCT TTC TTT GGT AGG-3’  Reverse: 5’- TTT CAA TAA ATC AGG TCC CAG G-3’ |
