# Supplementary material for: Lithium protects against paraquat neurotoxicity by NRF2 activation and miR-34a inhibition in SH-SY5Y cells
Source: Front Cell Neurosci. 2015 May 28;9:209. doi: 10.3389/fncel.2015.00209 (PMC4446540; doi:10.3389/fncel.2015.00209)
Supplement: Supplementary file 4 [file Image1.PDF]

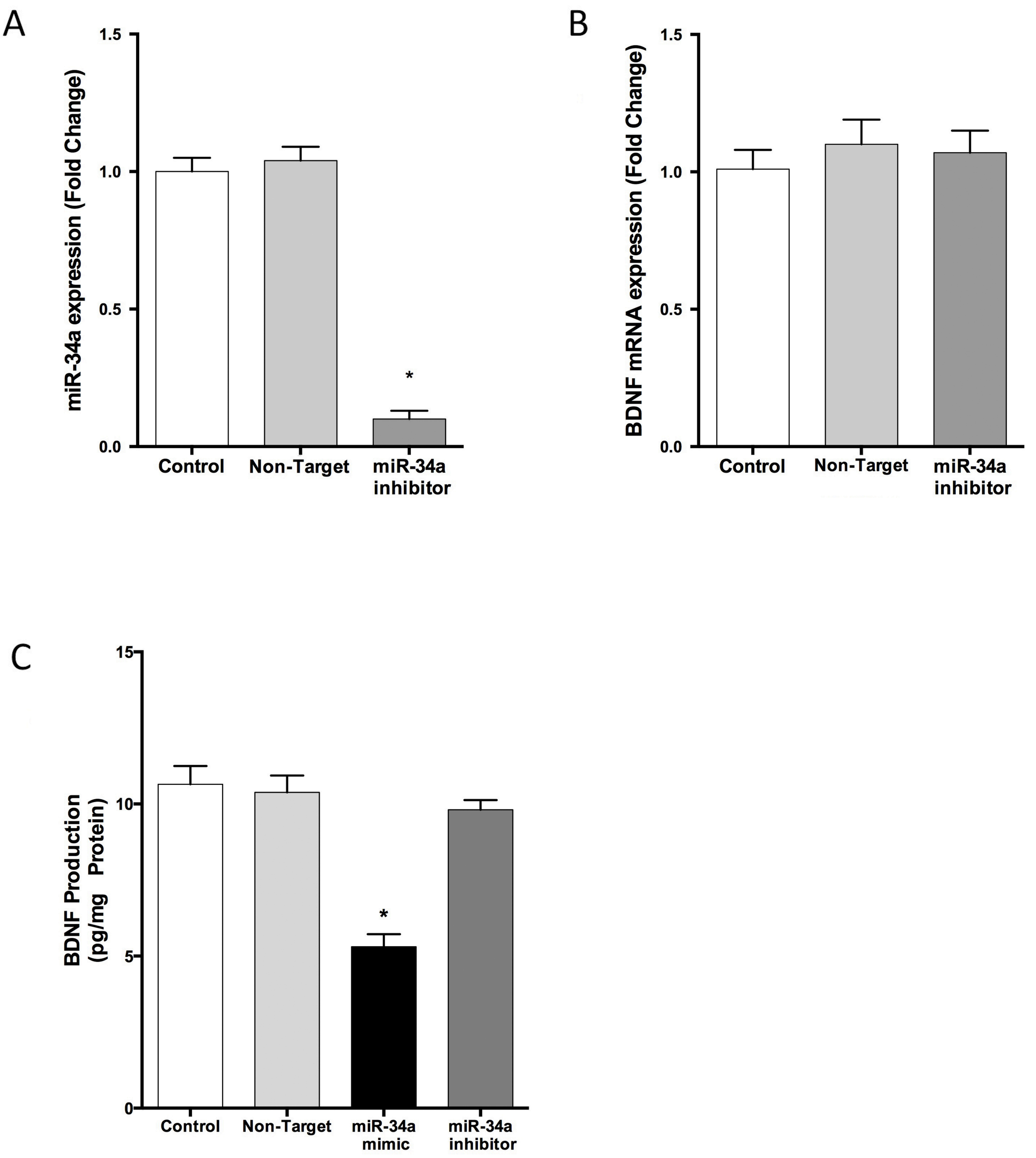

Supplementary Figure 1. Decreased expression of miR-34a via its inhibitor has no effects on BDNF mRNA expression and protein release. (A) SH-SY5Y cells were transfected with miR-34a inhibitor or non-target miRNA for 48 hours and collected for RNA isolation. The efficiency of transfection analyzed by qPCR. (B) BDNF mRNA level (predicted target of miR-34a) did not change after inhibitor transfection. (C) Secreted BDNF protein levels into culture medium were measured by BDNF ELISA kit. miR-34a overexpression decreased BDNF protein levels while miR-34a inhibitor did not alter BDNF protein level in SH-SY5Y cells. Control: cells without any transfection. The data are presented as mean ± S.E, n=5. (*$p<0.05$).
